# Is depression a disorder of electrical brain networks?

**DOI:** 10.1038/s41386-019-0511-8

**Published:** 2019-09-10

**Authors:** Yael Grossman, Kafui Dzirasa

**Affiliations:** 1 Department of Psychiatry and Behavioral Sciences, Duke University Medical Center, Durham, NC 27710 USA; 2 Department of Neurobiology, Duke University Medical Center, Durham, NC 27710 USA

**Keywords:** Emotion, Stress and resilience

Major depressive disorder (MDD) is one of the most prevalent and disabling neuropsychological disorders in the world, with 15% of adults expected to experience depression sometime in their lives. Current treatment options are largely ineffective, as only 50–70% of patients experience remission after multiple rounds of treatment [1]. Thus, there is a clear and immediate need for the development of novel therapeutics that prevent MDD. Nevertheless, this endeavor has been hampered by limited knowledge of the biology underlying the disorder.

A well-validated murine model of depression, chronic social defeat stress (CSDS) [2], can differentiate between mice that exhibit MDD-like behavior following stress exposure, termed “susceptible”, and those that do not, termed “resilient”. Our lab’s prior work exploring network dynamics linked to CSDS susceptibility found that susceptible mice exhibited greater prefrontal cortex (PFC)-dependent limbic synchrony [3]. Since susceptible and resilient mice experienced identical stress exposure but exhibited different network dynamics after CSDS, we hypothesized that differences in network dynamics exist prior to stress exposure and could serve as a biomarker for the vulnerable population of test mice (i.e., mice that *will* exhibit MDD-like behavior *following* future exposure to CSDS).

To test this hypothesis, multicircuit recordings during acute threat were collected from test mice before and after exposure to CSDS, and processed using discriminative cross-spectral factor analysis (dCSFA), a model of machine learning [4]. The dCSFA method was chosen for its interpretability (i.e., relatability to specific neural phenomena) and prediction (i.e., discrimination of behavioral variables). This approach identified four electrical network features, termed “electome factors”. These networks were validated using techniques previously demonstrated to increase vulnerability (e.g., early life stress, inflammation, and overexpression of the gene *Sdk1* in the ventral hippocampus). Only one of these electome factors, *Electome Factor 1 (EF1)*, was responsive to vulnerability manipulations and, consequently, validated as a network underlying vulnerability. Furthermore, techniques for treating susceptible mice after CSDS (e.g., ketamine administration and suppression of activity in PFC) did not have any significant effect on *EF1*, though these treatments suppressed other electomes associated with susceptibility. Activity in this network originates in the PFC and ventral striatum, relays through the amygdala and ventral tegmental area, and converges in the ventral hippocampus. Together, these results indicate that *EF1* is a biomarker of vulnerability and is distinct from MDD-like susceptibility.

Alternative techniques have identified networks indicative of individual vulnerability to social stress in rats [5]. Though vulnerability identification has not progressed to humans yet, recent functional magnetic resonance imaging studies in depressed patients have revealed distinct functional networks [6]. Furthermore, differences in functional connectivity successfully predict different subtypes of depression as well as responsiveness to treatment, suggesting that network-level analyses may provide an avenue for developing more successful treatments for depression.

Our findings demonstrate that network-level spatiotemporal dynamics can indicate previously obscured vulnerable individuals within heterogeneous populations. These results could support the development of novel therapeutic mechanisms targeted at preventing the emergence of MDD or encouraging resilience in vulnerable populations. Furthermore, they encourage exploration of electome networks that may signal other emotional states in health and disease.

## Funding and disclosure

NIH Grant R01MH120158 to K.D. The authors declare no competing interests.

## References

[1] Rush AJ (2006). Acute and longer-term outcomes in depressed outpatients requiring one or several treatment steps: a STAR*D report. Am J Psychiatry.

[2] Krishnan V (2007). Molecular adaptations underlying susceptibility and resistance to social defeat in brain reward regions. Cell.

[3] Hultman R (2016). Dysregulation of Prefrontal Cortex-Mediated Slow-Evolving Limbic Dynamics Drives Stress-Induced Emotional Pathology. Neuron.

[4] Hultman R (2018). Brain-wide Electrical Spatiotemporal Dynamics Encode Depression Vulnerability. Cell.

[5] Nakayama R, Ikegaya Y, Sasaki T (2019). Cortical-wide functional correlations are associated with stress-induced cardiac dysfunctions in individual rats. Sci Rep.

[6] Drysdale AT (2017). Resting-state connectivity biomarkers define neurophysiological subtypes of depression. Nat Med.

